# Assessing the Performance of a Computer-Based Policy Model of HIV and AIDS

**DOI:** 10.1371/journal.pone.0012647

**Published:** 2010-09-09

**Authors:** Chara E. Rydzak, Kara L. Cotich, Paul E. Sax, Heather E. Hsu, Bingxia Wang, Elena Losina, Kenneth A. Freedberg, Milton C. Weinstein, Sue J. Goldie, for the CEPAC Investigators

**Affiliations:** 1 Harvard Medical School, Boston, Massachusetts, United States of America; 2 Department of Health Policy and Management, Harvard School of Public Health, Boston, Massachusetts, United States of America; 3 Division of Infectious Diseases, Department of Medicine, Brigham and Women's Hospital, Boston, Massachusetts, United States of America; 4 Division of General Medicine, Department of Medicine, Massachusetts General Hospital, Boston, Massachusetts, United States of America; 5 Department of Orthopedic Surgery, Brigham and Women's Hospital, Boston, Massachusetts, United States of America; 6 Departments of Biostatistics and Epidemiology, Boston University School of Public Health, Boston, Massachusetts, United States of America; 7 Division of Infectious Diseases, Department of Medicine, Massachusetts General Hospital, Boston, Massachusetts, United States of America; University of Cape Town, South Africa

## Abstract

**Background:**

Model-based analyses, conducted within a decision analytic framework, provide a systematic way to combine information about the natural history of disease and effectiveness of clinical management strategies with demographic and epidemiological characteristics of the population. Among the challenges with disease-specific modeling include the need to identify influential assumptions and to assess the face validity and internal consistency of the model.

**Methods and Findings:**

We describe a series of exercises involved in adapting a computer-based simulation model of HIV disease to the Women's Interagency HIV Study (WIHS) cohort and assess model performance as we re-parameterized the model to address policy questions in the U.S. relevant to HIV-infected women using data from the WIHS. Empiric calibration targets included 24-month survival curves stratified by treatment status and CD4 cell count. The most influential assumptions in untreated women included chronic HIV-associated mortality following an opportunistic infection, and in treated women, the ‘clinical effectiveness’ of HAART and the ability of HAART to prevent HIV complications independent of virologic suppression. Good-fitting parameter sets required reductions in the clinical effectiveness of 1^st^ and 2^nd^ line HAART and improvements in 3^rd^ and 4^th^ line regimens. Projected rates of treatment regimen switching using the calibrated cohort-specific model closely approximated independent analyses published using data from the WIHS.

**Conclusions:**

The model demonstrated good internal consistency and face validity, and supported cohort heterogeneities that have been reported in the literature. Iterative assessment of model performance can provide information about the relative influence of uncertain assumptions and provide insight into heterogeneities within and between cohorts. Description of calibration exercises can enhance the transparency of disease-specific models.

## Introduction

Over the past fifteen years there has been remarkable progress in the treatment of HIV-1 infection.[1]–[4] Where highly potent combination antiretroviral therapy (HAART) is accessible, HIV has become a chronic treatable disease, albeit complex and costly, requiring lifelong management.[1], [5] There are a number of clinical and policy questions that remain to be addressed in HIV, ranging from the optimal time to begin antiretroviral treatment to how best to increase access to care and improve adherence to antiretroviral therapy. Unfortunately, no single study can include all possible strategies, and the rapid evolution in treatment options poses a challenge for trial-based investigations to keep pace with the questions to be answered. Even when clinical trials are conducted, they are often limited in their length of follow-up and rely on intermediate outcomes.[6]–[14]

Model-based analyses, conducted within a decision analytic framework, provide a systematic way to combine information about the natural history of disease, efficacy of different treatment regimens, and effectiveness of clinical management strategies with other relevant demographic and epidemiological characteristics of the target population.[15]–[17] When used within a decision-analytic framework, models can extend knowledge from empirical studies to other situations and can be used to evaluate alternative strategies not feasible to explore in a clinical trial. When the data are insufficient to support traditional forms of investigation, models offer a practical framework for managing uncertainty via sensitivity and “what-if” analysis.

Over the past 10 years, the “Cost-effectiveness of Preventing AIDS Complications (CEPAC)” model has been used to conduct analyses intended to guide HIV clinical decision-making and policy formulation in a variety of settings.[18]–[30] The model has been iteratively revised as new data become available, both about the disease itself, and the wide array of new treatment options. Persistent challenges with any simulation model of a complex disease include the detail required to reflect a realistic representation of the disease process, the pace at which data become available, and the need to continuously revisit assumptions in the context of new information. As the complexity of a model increases, so will the requirements for parameters. Input values are almost never available for all parameters, and analysts rely on approaches ranging from expert assumptions with careful sensitivity analyses to conducting calibration exercises that involve fitting model output to epidemiological data in order to inform uncertain parameter estimates. Regardless of the method used to parameterize the model, decision analysts seek to assess parameter uncertainty and to explore the relative influence of uncertain assumptions made. While sensitivity analyses to address parameter uncertainty are included in most decision analyses, analysts often conduct many exploratory analyses to assess the influence of model assumptions. When data are available to allow for such exercises, they provide an opportunity to assess the model's face validity and internal consistency. However, even when conducted, often these exercises are unable to be included in peer-reviewed publications due to space limitations.

In this paper, we describe a series of exercises that were conducted as we re-parameterized the CEPAC model to address clinical and policy questions in the United States relevant to HIV-infected women. This process required that data be extracted from the Women's Interagency HIV Study (WIHS) and adapted to a format required by the model.[31]–[34] We used this effort to assess the internal consistency of the model, identify influential assumptions on model outcomes, and assess the external consistency of the model with independent published analyses. This paper describes the process and steps taken to do so.

## Methods

The Cost-Effectiveness of Preventing AIDS Complications (CEPAC) model is a 1^st^-order (i.e., patient level) Monte Carlo simulation model of HIV disease, and has been previously described.[18]–[30] Disease progression in the model is characterized as a sequence of monthly transitions from one “health state” to another. Health states, descriptive of each patient's true underlying health, are defined by current and maximum HIV RNA, current and lowest CD4 lymphocyte count, and current and prior opportunistic infections. Drawing from an initial distribution of specified demographic (age, sex) and clinical characteristics (CD4 count, HIV RNA level, history of opportunistic infection), the model simulates a cohort of individual patients whose clinical course is tracked from model entry until death. A random number generator and a set of estimated probabilities are used to determine the sequence of clinical pathways that a given patient follows, while a running tally is maintained of all acute clinical events, the length of time spent in each health state, and the cost associated with each health state. Upon the patient's death, summary statistics for that individual are recorded. One million patients are simulated, one at a time, in order to provide stable estimates of long-term outcomes for each strategy. Model outcomes include intermediate outcomes such as number and type of opportunistic infections, time spent on treatment, and proportion alive each month, as well as long-term aggregate outcomes such as life expectancy, quality-adjusted life expectancy, and lifetime costs.

The progression of underlying HIV disease is modeled as a function of both HIV RNA and CD4 cell counts. Opportunistic infections are based on previous analyses of primary and published data, and are differentiated according to severity as previously described.[28], [31]–[35] Treatment with HAART and successful HIV RNA suppression result in a CD4 cell count rise, which in turn produces a reduction in the risk of acute opportunistic infections and death. HAART efficacy is modeled as an initial probability of virologic suppression and subsequent monthly probability of failure. For individuals on HAART who experience virologic failure, the CD4 cell count remains stable for a specified number of months, after which the CD4 cell count declines at a monthly rate governed by the current viral load. An independent protective effect of HAART is modeled as a multiplier which decreases the incidence of opportunistic infections and AIDS-related mortality in patients with virologic failure who remain on HAART (herein referred to as the *ART effect*).[36], [37]

Details regarding the analysis of data used in the CEPAC model may be found in prior publications [2]–[4], [18]–[30], [36]–[38]; in most of these analyses the natural history of disease progression in the absence of treatment was based on data from the Multicenter AIDS Cohort Study (MACS)—a longitudinal study of HIV/AIDS in gay and bisexual men initiated in 1984 and consisting of a cohort of over 5,600 men.[2]–[4], [35], [38]–[40] To address clinical and policy questions in the U.S. relevant to HIV-infected women, we extracted data from the Women's Interagency HIV Study (WIHS), a longitudinal study of HIV disease begun in 1993 and consisting of data from approximately 3,000 HIV-infected women and 1,000 uninfected women.[31]–[34] Using survival data from the WIHS, we established empiric calibration targets that included 24-month survival curves based on untreated HIV-infected women, according to starting CD4 cell count. These served as a comparison against which model-generated survival curves could be evaluated.

### Comparison of Natural History Model Projected Survival to Empiric Data

To compare natural history model projected survival with empiric data, we first generated Kaplan-Meier survival curves using the natural history model parameterized with data from the MACS. Second, we generated survival curves using the natural history model parameterized with new data from the WIHS. Third, we identified several uncertain assumptions relevant to natural history, which were varied extensively in sensitivity analysis. These assumptions, listed in order of subjective uncertainty, included the following: (a) the probability of “chronic AIDS” death (i.e., deaths occurring after 30 days of an OI diagnosis) is higher compared to the probability of AIDS death given no OI history (herein referred to as *attribution*); (b) the incidence of OIs and the probability of OI-related mortality change over time with disease progression (as defined by CD4 cell count and viral load) and differ by gender; (c) in the absence of HAART, the rate of CD4 decline is conditional on HIV RNA, and differs by gender. The parameters governing these assumptions were systematically varied and model-projected survival was compared with empiric 24-month survival from the WIHS. Consistency between model projections and empiric data was assessed by visually comparing the average model outcomes with the means and the 95% confidence intervals (CI) of the empiric survival data.

### Comparison of Treatment Model Projected Survival to Empiric Data

Survival data were obtained from women in the WIHS who initiated HAART between 1998 and 2002 and were followed for 24 months.[31]–[34] We assumed that HAART was not initiated until a woman's CD4 count reached 200/µl or less, and that 4 distinct HAART regimens were available.[41] We assumed that women who survived over the long-term would have had access to more recent regimens as they became available during their treatment, but would likely have experienced decreased efficacy due to previous exposure to other earlier HAART regimens. We explored a variety of scenarios designed to capture the heterogeneities of treatment response given different levels of previous exposure to non-HAART monotherapy or combination antiretroviral therapy for women in the WIHS cohort, as well as differences in adherence and loss to follow-up. Kaplan-Meier survival curves were constructed from model simulation outputs and visually compared to WIHS empirical survival curves for HIV-infected women receiving HAART. In addition, the goodness of fit was quantitatively evaluated by comparing the sum of the absolute differences between model estimates at 6, 12, 18 and 24 months with the mean empiric data at the same time points. Parameter sets were then ranked based on the value of the sum of the absolute differences for all of the time points; those parameter sets with the lowest values (i.e., smallest difference compared to the empiric data) were considered to be more consistent with the empiric survival data.

We explored the influences of changes in assumptions and treatment parameter values on the consistency between the model-projected survival and the empiric data. We identified several key uncertain treatment-related parameters and assumptions which we varied systematically, first one at a time and then in combination, to assess their impact on model-projected survival (**Table 1**). These included: (a) ‘clinical effectiveness’ of HAART (a function of regimen efficacy, tolerance without major toxicity, adherence, and personal choice to remain on treatment); (b) the magnitude of an independent protective effect of HAART on opportunistic infection incidence and AIDS mortality in patients with virologic failure (herein referred to as the *ART effect*); (c) monthly CD4 cell gains while on effective HAART; (d) the risk of early and late treatment failure (where “early treatment failure” is defined as regimen failure within the first 6 months of treatment with a specific regimen while, “late treatment failure” is defined as the monthly probability of treatment failure after initially successful virologic suppression after the first 6 months of treatment); (e) estimates of the delay in CD4 count decline following virologic rebound associated with HAART failure; and (f) the maximum duration of treatment efficacy in patients who did not experience virologic failure.

**Table 1:** Summary of selected treatment parameter values.

Variable or Assumption	Initial Value	Exploratory Range
HAART Efficacy (% Viral Load Suppression at 24 weeks)		
EFV + AZT + 3TC	75%	+/− 10% to 90%*
IDV + AZT (or d4T) + 3TC	60%	(10% increments)
LPV/r + TDF + FTC + AZT	61%	
ENF + OBR	32.7%	
OBR (2 PIs + 2 NRTIs)	15%	
Mean CD4 Gain/µl on Successful Treatment over first 12 months (SD)		
Time Period 1 (months 1–2)		
EFV + AZT + 3TC	68.81 (17.20)	+/− 20% to 50%
IDV + AZT (or d4T) + 3TC	25.02 (6.26)	
LPV/r + TDF + FTC + AZT	68.71 (17.18)	
ENF + OBR	75.63 (18.91)	
OBR (2 PIs + 2 NRTIs)	26.04 (6.51)	
Time Period 2 (months 3–12)		
EFV + AZT + 3TC	3.60 (0.90)	+/− 20% to 50%
IDV + AZT (or d4T) + 3TC	1.31 (0.33)	
LPV/r + TDF + FTC + AZT	3.60 (0.90)	
ENF + OBR	3.96 (0.99)	
OBR (2 PIs + 2 NRTIs)	1.36 (0.34)	
Late Treatment Failure*		
Pooled Monthly Probability	0.021099	None, 50% decrease to 200% increase
ART Effect†		
CD4 <50/µl	0.78	No ART effect, 0.78, 0.66, 0.54, 0.25
CD4 >50/µl	0.66	No ART effect, 0.78, 0.66, 0.54, 0.25
Assumptions		
Delay in CD4 decline	1 year	None, 2 years
Force failure	10 years	5, 15 and 20 years, Never

3TC  =  lamivudine; ART  =  antiretroviral therapy; AZT  =  zidovudine; ddI  =  didanosine; d4T  =  stavudine; EFV  =  efavirenz; ENF  =  enfuvirtide; FTC  =  emtricitabine; HAART  =  highly active antiretroviral therapy; IDV  =  indinavir; LPV/r  =  lopinavir/ritonavir; NRTI  =  nucleoside reverse transcriptase inhibitors; NNRTI  =  non-nucleoside reverse transcriptase inhibitors; OBR  =  optimized background antiretroviral regimen; SD  =  standard deviation; TDF  =  tenofovir.

*Note that treatment efficacy was capped at a maximum of 95% and a minimum of 5% regardless of regimen; in some instances this resulted in a percent change in efficacy that was less than the original stated change.

† The ART effect is defined as an independent protective effect of HAART and is modeled as a multiplier which decreases the incidence of opportunistic infections and AIDS-related mortality in patients with virologic failure who remain on HAART.

### Assessment of Consistency with Independent Analyses of Data from the WIHS

By calibrating to cohort-specific data, we implicitly assumed the ‘clinical effectiveness’ of HAART reflects several factors (e.g., regimen efficacy, tolerance, and adherence). We assessed the consistency of the calibrated model to independent analyses that used a distinct subset of data from the WIHS not used in the initial parameterization. We identified a published analysis[34] that provided estimates of time on treatment and time to regimen switch from women treated with HAART in the WIHS cohort; in that study, ‘switching’ was defined to include participants who discontinued or switched to a less intense regimen as well as those switching to a different HAART regimen for any reason.[34] Using the calibrated model we then conducted simulations with the 50 best-fitting parameter sets to compare the estimated median time on treatment and time to regimen switch with these published cohort-specific data.[34]

Finally, to gain insight into the nature of the differences between the WIHS cohort[34] and the clinical trials from which we obtain treatment efficacy data to use in contemporary analyses, we conducted simulations using the baseline model prior to calibration, and tracked all women who switched from a given regimen due to virologic failure as well as women who experienced either minor or nonfatal major toxicity. We compared the model-generated estimated ‘switching’ (from either virologic failure or single drug switch due to intolerance or toxicity) to the published estimates of time on treatment and time to regimen switch reported from the WIHS (Kirstein et al.),[34] after removing women who discontinued HAART, to make the model-generated estimate of ‘switching’ more comparable. For this exercise we assumed that approximately 1 in 4 women with minor toxicity would discontinue treatment.[42]

#### Influential Factors on Projection of Long-Term Outcomes

We compared the differences between estimates of life expectancy derived from the model calibrated to the 24-month short-term data (using the mean of the 50 best-fitting sets) to those generated using the original model parameters prior to calibration. We also explored uncertain variables hypothesized to be influential on long-term outcomes, including probability of late treatment failure (defined as the monthly probability of treatment failure after initially successful virologic suppression), an independent protective effect of HAART on mortality in patients with virologic failure (i.e., *ART effect*), delay in CD4 decline following virologic rebound associated with HAART failure, availability of 5 sequential lines of HAART rather than 4 lines, and major HAART toxicity.

### Data

Cohort characteristics and natural history parameter values for HIV-infected women in the WIHS cohort who did not receive HAART are provided in **Supporting Information S1.** [31] Natural history inputs estimated from the WIHS dataset were derived using similar linear interpolation methods as those used to develop analogous estimates for the MACS dataset.[31]–[34] Corresponding data are provided for the MACS cohort in **Supporting Information S1**.[2]–[4], [35], [38]–[40]

The definition of HAART was based on guidelines from the Department of Health and Human Services/Kaiser and the International AIDS Society—USA Panel guidelines.[1], [34], [41] Women were considered to be on HAART if their regimen consisted of one of the following: “two or more nucleoside reverse transcriptase inhibitors (NRTIs) in combination with at least one protease inhibitor (PI) or one non-nucleoside reverse transcriptase inhibitor (NNRTI); one NRTI in combination with at least one PI and at least one NNRTI; a regimen containing ritonavir and saquinavir in combination with one NRTI and no NNRTIs; or an abacavir-containing regimen of three or more NRTIs in the absence of both PIs and NNRTIs.” [1], [34], [41]

HAART regimens used in this analysis are representative of those available during the treatment era between 1998 and 2002 during which a subset of women in the WIHS initiated treatment; these regimens are based on those described by Walensky and colleagues.[43] HAART efficacy estimates were based on a threshold of suppression of HIV RNA <400 copies/µl at 24 weeks after initiation of a given HAART regimen. A threshold of <400 copies/µl was used, as this reflected the minimum threshold level of virus detectable by most tests used during that timeframe.[38], [44], [45] Efficacy estimates used intent-to-treat data for all regimens.[42], [46]–[49] Estimates of total mean CD4 count gains while on specific HAART regimens incorporated loss-to-follow up in the cohort.[42], [46]–[49] **Supporting Information S1** provides assumptions about HAART efficacy, OI prophylaxis efficacy and risk of toxicity. [33], [42], [46]–[58]

Estimates of regimen-specific monthly probabilities of late failure after initial successful virologic suppression were calculated using efficacy estimates for 24 weeks and the percent suppressed at the furthest reported time point after 24 weeks (usually 48 or 96 weeks). Regimen-specific late failure probabilities were then used to calculate the pooled probability of late regimen failure after initial successful suppression.[42], [46]–[49] Estimates of the ART effect were based on values reported by Kousignian and colleagues.[50] Individuals with a CD4 count <50/µl had an ART effect value of 0.78 while those with a CD4 count ≥50/µl had an ART effect value of 0.66; these amounted to a decrease in the magnitude of risk of 22% and 34%, respectively.[50] The plausible range explored included no ART effect (no risk reduction), an ART effect of 0.54 based on a study by Cole et al. (46% reduction in the probability of OI's and chronic AIDS death), and an ART effect of 0.25 (75% reduction in risk).[51]

## Results

### Performance of the Natural History Model

**Figure 1:**
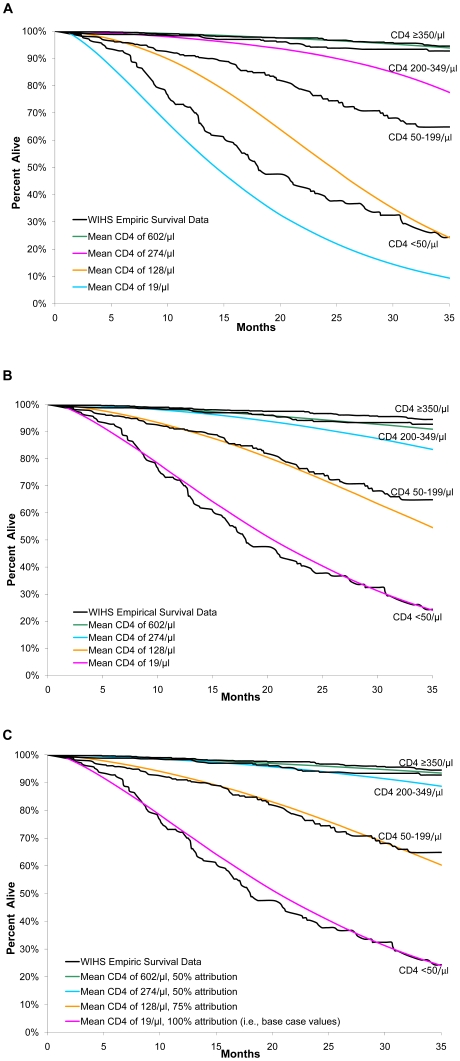
Base case natural history data with WIHS cohort characteristics. Part A of Figure 1 shows the model-estimated survival of the WIHS cohort using natural history input parameters derived from the MACS. With the exception of the highest CD4 stratum (CD4 ≥350/µl), the model underestimates survival for individuals with initial CD4 cell counts <350/µl, particularly as follow-up time increased. Part B of Figure 1 shows the base-case model-estimated survival using natural history input parameters derived from the WIHS. The re-parameterized model more closely approximates the empiric data for the three lowest CD4 strata (generally within the 95% CI), although the model continues to marginally underestimate mean survival in CD4 strata CD4 50–199/µl and CD4 200–349/µl. Model-projected survival in the highest CD4 stratum (≥350/µl) is more significantly underestimated, with a better visual fit achieved using natural history inputs derived from the MACS (Figure 1, Part A). Part C of Figure 1 shows the impact of adjustment of CD4-specific attribution on model-projected survival. Part C of Figure 1 demonstrates that better consistency between model-projected survival and empiric data was best achieved with adjustment of CD4 stratum-specific attribution. Specifically, reduction of the incrementally increased probability of AIDS-related mortality in patients with a history of previous opportunistic infections (*attribution*) by 25% for CD4 50–199/µl and 50% for CD4 strata ≥200/µl resulted in better estimation of the empiric survival data.

**Figure 1**, **Part A**, shows the model-estimated survival of those members of the WIHS cohort who did not receive HAART, using natural history input parameters derived from the MACS. With the exception of the highest CD4 stratum (CD4 ≥350/µl), the model underestimated survival for individuals with initial CD4 cell counts <350/µl, particularly as follow-up time increased.

**Figure 1**, **Part B**, shows the model-estimated survival using natural history input parameters derived from the WIHS. The re-parameterized model more closely approximates the empiric data for the three lowest CD4 strata (generally within the 95% CI) although the model continues to marginally underestimate mean survival in CD4 strata 50–199/µl and 200–349/µl. Model-projected survival in the highest CD4 stratum (≥350/µl) is more significantly underestimated, with a better visual fit achieved using natural history inputs derived from the MACS.

Better consistency between model-projected survival and empiric data was achieved with adjustment of CD4 stratum-specific attribution. Specifically, incrementally reducing the probability of AIDS-related mortality in patients with a history of previous opportunistic infections (*attribution*) by 25% for CD4 50–199/µl and 50% for CD4 ≥200/µl resulted in better estimation of the empiric survival data (**Figure 1**, **Part C**). These adjusted values for attribution remained within 95% CI of the original estimates. Enhanced consistency between model-projected survival and empiric survival was not achieved with only changes in OI incidence or plausible changes in CD4 cell decline.

### Performance of the Treatment Model

For members of the WIHS cohort who received HAART, model-projected survival over 12 months and at 24 months was higher than the mean empiric survival. An initial exploratory set of one-way sensitivity analyses were conducted to provide insight into the magnitude of influence of each uncertain assumption. The most influential of these one-way sensitivity analyses included reductions in the (1) ‘clinical effectiveness’ of HAART (a function of regimen efficacy, tolerance, adherence, and personal choice to remain on treatment); (2) CD4 cell gain on HAART; and (3) ART effect. None of the one-way sensitivity analyses achieved simultaneous consistency with both 12- and 24-month outcomes (**Supporting Information S1** provides a summary of changes in ‘clinical effectiveness’). In general, scenarios most consistent with the empiric data at 12 months underestimated survival at 24 months, while those most consistent with the empiric data at 24 months overestimated survival at 12 months.

Using insights from the one-way sensitivity analyses, a series of additional multi-way sensitivity analyses allowed assumptions to vary by regimen (e.g., 50% decrease in CD4 cell gain on 1^st^ and 2^nd^ line HAART but an increase in CD4 cell gain for 3^rd^ and 4^th^ line HAART) and also allowed changes in two or more variables simultaneously (e.g., 50% reduction in ‘clinical effectiveness’ of HAART and 50% decrease in CD4 gain). Selected results are shown in **Supporting Information S1**. In general, multi-way sensitivity analyses allowed less extreme (and more plausible changes) in individual variables while providing better visual fits to the data.

**Figure 2:**
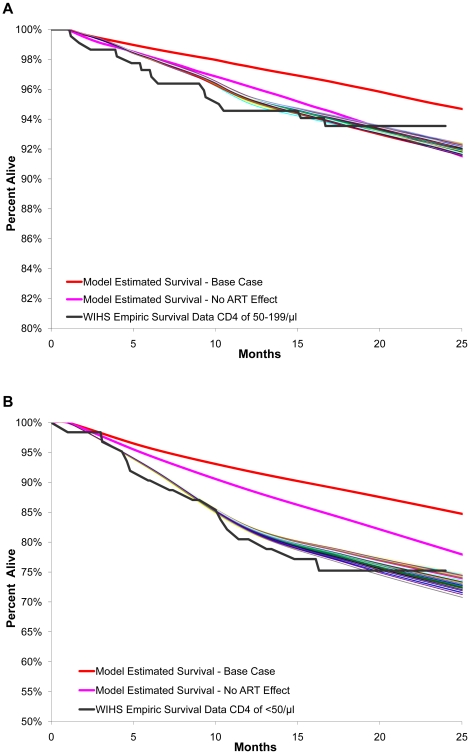
Top 50 best fits of WIHS empiric survival for CD4 50–199/µl and CD4 <50/µl. Part A of Figure 2 illustrates the top 50 best fits of the empiric survival data for CD4 50–199/µl. Nearly all runs in the top 50 combined changes in both ‘clinical effectiveness’ of HAART (a function of regimen efficacy, side effects or toxicity, adherence, and personal choice to remain on HAART) and estimates of CD4 gains while on effective HAART. The majority of the 50 best fits had a 2- to 3-fold increase in the rates of failure/switching/discontinuation of early lines of HAART in combination with an increase of 40%–75% in treatment efficacy in later lines of HAART. Part B of Figure 2 illustrates the top 50 best fits of the empiric survival data for CD4 <50/µl. Nearly all runs in the top 50 combined changes in both ‘clinical effectiveness’ of treatment (a function of regimen efficacy, side effects or toxicity, adherence, and personal choice to remain on HAART) and estimates of CD4 gains while on effective HAART. The majority of the 50 best fits had a 2- to 3-fold increase in the rates of failure/switching/discontinuation of early lines of HAART in combination with an increase of 30%–75% in treatment efficacy in later lines of HAART.

Varying each of the uncertain assumptions individually, in combination, and according to HAART regimen, generated more than 1500 unique combinations of parameters for each CD4 stratum. Including all 1500 unique combinations, we calculated the absolute difference between the mean 6, 12, 18 and 24 month model-projected and empiric survival. For each of the CD4 strata, we selected the 50 parameter sets with the smallest absolute difference between the mean of the empiric data and the mean of the projected model outcomes at 6, 12, 18 and 24 months. Herein we refer to these 50 parameter sets as the “best-fitting” parameter sets. **Figure 2** shows the model-projected 24-month survival for the best-fitting sets versus the empiric data for CD4 50–199/µl (**Figure 2**, **Part A**) and CD4 <50/µl (**Figure 2**, **Part B**).

For CD4 50–199/µl (**Figure 2**, **Part A**), the 50 best-fitting parameter combinations that produced the best estimates of 6, 12, 18 and 24 month survival (i.e., minimized the difference between model output and empiric survival across all four time points) were similar, in that the ‘clinical effectiveness’ of 1^st^ and 2^nd^ line HAART was reduced, while that of 3^rd^ and 4^th^ line HAART was increased in combination with similar directional changes in CD4 gain estimates. Specifically, across the 50 best-fitting parameter sets, there was a 2- to 3-fold increase in failure or discontinuation rate for HAART lines 1 and 2, corresponding to a 70% reduction in the average overall virologic suppression in the cohort. Note that this average overall virologic suppression corresponds to that of a heterogeneous cohort; there are some members who are non-adherent, some who elect to change to a less efficacious non-HAART regimen, and some who discontinue HAART. In contrast, across the 50 best-fitting parameter sets, the clinical effectiveness of 3^rd^ and 4^th^ line HAART was increased by 40% to 75%. In the majority of best-fitting parameter sets, CD4 gain was reduced by 20% to 50% for 1^st^ and 2^nd^ line HAART, while CD4 gain was increased by 20% to 50% for lines 3 and 4. Compared to previous one-way analyses, runs using combinations of less extreme value changes across multiple parameters provided improved fits of the empiric data. For CD4 <50/µl (**Figure 2**, **Part B**) the 50 best-fitting parameter combinations that produced the best estimates of 6, 12, 18 and 24 month survival were similar to those in the higher CD4 strata.

### Assessment of Consistency with Independent Analyses of Data from the WIHS

Using the model calibrated to the WIHS cohort, we estimated percentage who switched from their initial 1^st^ and 2^nd^ line HAART regimens within one year and the estimated median time to 3^rd^ line HAART; using the 50 best-fitting parameter sets. Model-projected ‘switching’ (1^st^ or 2^nd^ line HAART) was 65.8% in the first year, and the estimated median time to 3^rd^ line HAART was 28.3 months. In comparison, Kirstein et al.[34] reported that among women initiating HAART in the WIHS cohort, 65% (95% confidence interval [CI]: 62%, 68%) ‘switched’ (for any reason) their initial HAART regimen within one year of initiation, and that the median time on 1^st^ and 2^nd^ line HAART was 26 months (**Table 2**, **left side**).[34], [42]

**Table 2:** Comparison of model-generated estimates of HAART regimen ‘switching’ versus published data.

	Estimated “switching” in the model calibrated to the WIHS cohort	Estimated “switching” in the baseline model
Functional definition of “switching”	Switch to a different HAART regimen, any drug change, discontinuation, or switch to a less intense therapy	Switch to a different HAART regimen or any single drug change in current HAART regimen

	Kirstein et al*	Model†	Kirstein et al*	Model‡
Percent switched in 1 year	65%	65.8%	21.4%–45.6%	25%–45.25%

HAART  =  highly active antiretroviral therapy; WIHS  =  Women's Interagency HIV Study.

*Extracted from “Timing and Characteristics of Switching HAART Regimens in WIHS between 1994 and 2000” (Kirstein et al. JAIDS 2002).[34]

† Average of 50 best-fitting parameter sets weighted by CD4 cell distribution.

‡ Lower bound of 20% represents proportion switching 1^st^ line HAART due to virologic failure; upper bound of 45.25% represents proportion switching 2^nd^ line HAART due to virologic failure plus an assumption that 25% of women with minor toxicity and all women with nonfatal major toxicity require a single drug switch. This estimate is based on data from Staszewski et al showing that of the 43% who discontinued indinavir plus two nucleoside reverse transcriptase inhibitors for any reason, 11% was due to symptoms such as gastrointestinal effects.[42]

Using the baseline model prior to calibration, the estimated switching as a result of virologic failure with 1^st^ and 2^nd^ line HAART was 25% to 40%; if we assumed 25% of women who experienced minor toxicity and all women with nonfatal major toxicity required a single drug change, the model-generated “estimated switching” ranged from 28.25% to 45.25%. These results were consistent with the published data; when we excluded women who discontinued HAART, Kirstein reported between 21.4% and 45.6% experienced a ‘switch’ in their HAART regimen (**Table 2**, **right side**).[34], [42]

### Projected Long-Term Outcomes

**Figure 3:**
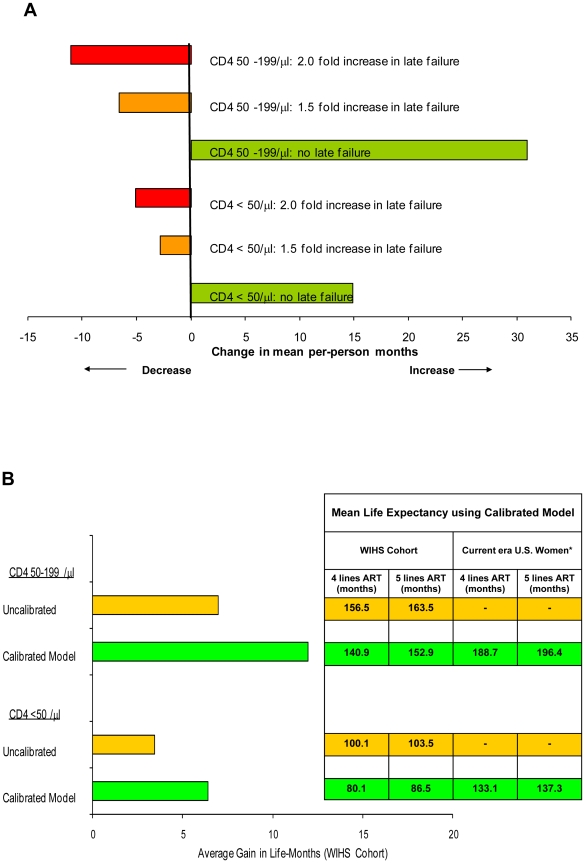
Impact of alternative assumptions on life expectancy. Results from sensitivity analyses showed that the most influential variable on long-term outcomes was the probability of late treatment failure. Part A of Figure 3, shows the impact of varying our base case assumptions (0.021) from no late failure to a 2-fold increase in late failure. Depending on the baseline CD4 cell count, life-expectancy was increased by 14.8 to 30.9 months with no late failure (green), and was decreased by 2.8 to 6.6 months with 1.5-fold increase in late failure (orange), and by 5.1 to 11.0 months with a 2-fold increase in late failure (red). The magnitude of these changes was similar regardless of whether we assumed 4 lines or 5 lines of HAART. Part B of Figure 3 shows the impact of 5 lines of HAART versus 4 lines of HAART on life expectancy. The average life expectancy projected by the model calibrated to the 24-month short-term data (using the mean of the 50 best-fitting sets) was 140.9 months (range, 130.5-148.4 months) among the patients with CD4 50–199/µl and 80.1 months (range, 65.9–87.3 months) among those with CD4 <50/µl. Average life-expectancy projected by the uncalibrated model varied with different assumptions about the ART effect, ranging from 123.3 months (no ART effect) to 156.5 months (ART effect) in patients with CD4 50–199/µl, and from 73.2 months (no ART effect) to 100.1 months in patients with CD4 <50/µl (ART effect). Figure 3, Part B, shows the incremental gains provided by 5 lines of HAART versus 4 lines of HAART were greatest using the calibrated model (green bars), and lowest using the uncalibrated model assuming no ART eff ect (orange bars).

The life expectancy projected by the cohort-specific model calibrated to the 24-month short-term data (using the mean of the 50 best-fitting sets) was 140.9 months (range, 130.5–148.4) among the patients with CD4 50–199/µl and 80.1 months (range, 65.9–87.3) among those with CD4 <50/µl assuming a mean cohort age of 34 years. The most influential variable on long-term outcomes in our simulation of the WIHS cohort was the probability of “late treatment failure,” defined as the monthly probability of treatment failure after initially successful virologic suppression. **Figure 3**, **Part A**, shows the impact of varying our base case assumptions (probability of late failure, 0.021) from no late failure to a 2-fold increase in late failure. Depending on the baseline CD4 cell count, life expectancy was increased by 14.8 to 30.9 months with no late failure, and was decreased by 2.8 to 6.6 months with a 1.5-fold increase in late failure, and by 5.1 to 11.0 months with a 2-fold increase in late failure.

**Figure 3**, **Part B**, shows that when simulating the WIHS cohort, the incremental gains provided by 5 lines of HAART compared to 4 lines were greater using the calibrated model. To estimate the life expectancy that would be expected in HIV-infected women today in the U.S., we used the calibrated natural history model to superimpose contemporary treatment strategies utilizing sequential lines of highly efficacious HAART. Assumptions made about efficacy and tolerability of contemporary HAART are provided in **Supporting Information S1**.[46]–[48], [52]–[55], [59]–[63] Projected life-expectancy in HIV-infected women on contemporary regimens that are currently available ranged from 133.1 to 188.7 months given 4 lines of therapy, and 137.3 to 196.4 months given 5 lines of therapy, depending on the CD4 cell count and assuming a mean cohort age of 34 years (**Figure 3**, **Part B**, **embedded table**).

## Discussion

This paper provides a description of the initial iterative process we utilized to assess model performance and gain insight about the generalizability of analyses relying on data derived from particular study cohorts.

Model-estimated survival of the WIHS all-female cohort using natural history input parameters derived from the MACS all-male cohort underestimated survival for individuals with initial CD4 cell counts <350/µl, particularly as follow-up time increased. Using data from the WIHS, coupled with moderate changes in mortality for those with a history of OI for the two highest CD4 strata, the re-parameterized model closely approximated the empiric data, demonstrating good internal consistency. While the differences between model survival estimates using MACS versus WIHS-derived parameter values could theoretically reflect gender differences in natural history, prior data suggests that cohort differences distinct from gender, such as underlying differences in general health status and co-morbidities are more likely to explain differences in estimates.[2]–[4], [31]–[35], [38]–[40]

Comparison of model-estimated survival of women on HAART with empiric WIHS survival data showed the model overestimated short-term survival. Adjustment of influential treatment assumptions (e.g., ‘clinical effectiveness’, the ART effect and the CD4 gain on treatment) individually across all lines of HAART did not produce a good fit to either 12- or 24-month survival. In contrast, scenarios that reduced the ‘clinical effectiveness’ of earlier treatment regimens and increased that of later regimens (e.g., 3^rd^ and 4^th^ line HAART), more closely approximated the empiric published data. Further, multi-way sensitivity analyses that simultaneously varied these assumptions allowed less extreme (and more plausible) changes in individual variables while providing better visual fits to the published data.

Examination of the good-fitting parameter sets to the empiric data revealed several interesting observations. First, for both CD4 count strata, good fits to the data required that the ‘clinical effectiveness’ of 1^st^ and 2^nd^ line HAART be reduced such that the “implied failure rates” were 2.0 to 3.5 fold higher. Importantly, as described above, we considered ‘clinical effectiveness’ as a proxy for the net impact of regimen efficacy, tolerance without major toxicity, adherence, and personal choice to remain on treatment. Accordingly, the “implied failure rate” associated with the model calibrated to the WIHS cohort serves as a proxy for virologic failure, toxicity or side effects leading to a change in regimen, and discontinuation of HAART for undocumented reasons. In contrast, for both CD4 count strata, best fits to the data were obtained with a 40% to 60% increase in the effectiveness of 3^rd^ and 4^th^ line HAART, with analogously lower failure/discontinuation rates.

The more than 50% reduction in ‘clinical effectiveness’ that characterized the best fitting parameter sets is inconsistent with the higher treatment efficacy documented in more recent studies, [46]–[48], [52]–[55], [59]–[63] the data used in this exercise were based on a specific cohort from 1998 and 2002 and would not be expected to reflect more recent care patterns and improved outcomes. Further, while we used intention to treat efficacy data from clinical trials for our initial parameterization, the proportion who choose to change regimens or stop therapy in clinical trials may be lower than in cohort studies such as this one.[42], [46]–[49]

Recognizing that newer data show better tolerated regimens and higher treatment efficacy, the necessity for such high failure rates in initial regimens to calibrate the model prompted us to consider the particularities of this specific cohort, their clinical histories and past ART experience, as well as their behaviors including adherence, discontinuation of HAART, and choices about continued treatment following HAART toxicity. We concluded that the substantial reduction in ‘clinical effectiveness’ with 1^st^ and 2^nd^ line HAART regimens in this historical simulation could very well be plausible, given that only 16% to 20% of women were completely ART naïve prior to HAART initiation; approximately 80% had some previous exposure to ART through mono- or combination therapy.[33], [34] Furthermore, 44%–48% of women who initiated treatment had a diagnosis of AIDS, suggesting very advanced disease. In contrast to the reduction in ‘clinical effectiveness’ for 1^st^ and 2^nd^ line HAART required to calibrate the model to the WIHS, the efficacy of 3^rd^ and 4^th^ line HAART required an increase that ranged from 30% to 75%; this considerable increase in efficacy is likely attributable to both the availability of new and more effective treatment regimens and an increasingly homogeneous group of women more likely to pursue, adhere to, and continue treatment.

It is notable, although not unusual for the time period, that a sizable proportion of women in the cohort elected to discontinue HAART. For example, between April 1997 and September 1997, when many women had initiated HAART, 45.6% of these women switched regimens and 18% reported discontinuing HAART (13% switched to a less intensive regimen and 5% discontinued therapy completely).[34] By three years later, in September 2000, the percentage discontinuing therapy completely increased from 5% to 11.4%.[34] Similar rates of discontinuation have been seen in both clinical trials and in cohort studies. For example, Staszewski et al reported 27%–43% discontinuation of HAART unrelated to efficacy in a clinical trial of indinavir plus two nucleoside reverse transcriptase inhibitors versus efavirenz plus two nucleoside reverse transcriptase inhibitors.[42] Hammer reported that the overall rate of premature discontinuation was 20% in a clinical trial comparing zidovudine (or stavudine) and lamivudine (28%) versus indinavir, zidovudine (or stavudine), and lamivudine (12%).[49] Several cohort studies described a high rate of discontinuation and short median duration of time on a specific regimen. Saag et al. described the increasing number of unique antiretroviral regimens between 1988 and 1998 and a median duration of a specific regimen of 4 months.[64] Van Roon et al. reported that 25% of their clinic patients discontinued HAART within 1 year of initiating therapy.[65] An Italian cohort found that 36% of men who began HAART modified or discontinued their initial regimen over a median follow-up time of 11 months.[66] Mocroft et al. estimated that 26% of their patients initiating HAART modified or discontinued their regimen within 6 months of initiation and that 45% had modified or discontinued their regimen after a median follow-up time of 14 months.[67]

The life expectancy projected by the model calibrated to the 24-month short-term cohort-specific data was 140.9 months using the mean calculated from simulations using the 50 best-fitting parameter sets (with individual estimates of the 50 best-fits ranging from 130.5–148.4 months) among the patients with CD4 50–199/µl. Further, the incremental gains projected by 5 lines of HAART versus 4 lines of HAART using the empirically calibrated model (**Figure 3**, **Part B**) were twice those predicted by the model prior to calibration. We also found that uncertain assumptions, such as late failure, while not influential on short-term outcomes, exerted a major impact on the predicted life expectancy. While estimates of life expectancy varied considerably with plausible changes in uncertain assumptions, the incremental gains associated with comparing different treatment strategies within a single cohort varied far less. The implication is that results of incremental cost-effectiveness analyses, for example those conducted to inform choices among competing treatment options, may be less affected by this variation; in contrast, analyses that seek to project long-term estimates of life expectancy or cost for a population of HIV-infected persons, may be more variable.

Our analysis has several important limitations. First, this analysis is not intended to depict a formal empirical calibration process. Rather, this paper was intended to provide a description of the “real world” iterative process of assessing model performance while building a simulation model of a complex disease. In addition, we sought to demonstrate the kind of insights that can be obtained by this type of exercise while providing a description that is intended to increase the transparency of a model development phase. Although we intended to explore the comparative implications of using WIHS versus MACS cohort data, our primary goal was not to fit the model to empiric data. In fact, we would not want to use a model empirically calibrated to older data, reflecting much lower treatment efficacy, to inform current policy questions that could contribute to decisions in the future. Furthermore, we recognize that there are alternative methods for sampling the parameter space including utilization of Bayesian methods, random sampling or complex optimization algorithms. Our guided approach was chosen after careful consideration of the practical and theoretical strengths and limitations of these alternatives, given our goal was to conduct an exploratory exercise; that being said, it is possible we did not sufficiently explore the entirety of the parameter space. These exercises can play an important role in characterizing the effects of key uncertain assumptions, identifying logical inconsistencies, and helping the analyst to understand and describe the performance of the model.

Second, cohort heterogeneities pose challenges to assessing model performance in that it is impossible to reflect all patient and population level differences in any analysis; the availability of data that adequately characterize heterogeneities within this study cohort remain limited. Some differences between the WIHS cohort and the clinical trial cohorts used to generate initial HAART efficacy estimates[42], [49] are clear; for example, the WIHS is all women (versus trials often with more than 80% male), more than 30% report a history of injection drug use (versus only 10–18% in trials), and nearly two-thirds are black or Hispanic (versus more than 50% white in many trials).[33], [34] Furthermore, heterogeneities in prior treatment exposure, underlying health status, patient adherence, and patient preferences about treatment, could have substantial effects on outcomes which must be taken into consideration; these and other unknowable factors could have directly or indirectly contributed to the high rates of switching and discontinuation of early lines of HAART in women in the WIHS. For example, toxicities have been reported as an important reason for discontinuation of therapy,[66] and a study by Ahdieh and colleagues reported that women were twice as likely as men to discontinue HAART because of toxicities.[68]

Third, treatment regimens could not be simulated with complete accuracy. Between the period of April 1996 and September 1996 there were roughly 13 unique HAART regimens used in the WIHS, with 25% of women taking the most common regimen which consisted of zidovudine, lamivudine and indinavir.[34] However, by the year 2000, there were 171 unique HAART regimens reported in the cohort, with fewer than 15% of women taking the most common regimen of stavudine, lamivudine and nelfinavir.[34] We attempted to account for HAART era effects on treatments used by using values representative of commonly-used regimens for the given time period during which the WIHS treatment data were collected.[43] However, we recognize these assumptions were at best approximations of the actual range of regimens used.

We emphasize that this analysis is not intended to be a representation of the current treatment environment, where there have been substantial improvements over time in response to treatment, both in terms of drug efficacy and reductions in treatment failure, in addition to decreases in drug toxicity.[59], [60], [62], [63], [69] Rather, the purpose of these exercises was to assess whether the model could produce results consistent with the data used to parameterize the model (i.e., internal consistency and validity), and could simulate a specific cohort such that outcomes were consistent with independent data from that cohort. Using this same model to simulate access to contemporary treatment strategies in HIV-infected women in the United States today, we found the projected life expectancy in women with a mean CD4 cell count of 350/µl, exceeded 250 months (>21 years) given 5 lines of therapy and assuming initiation of HAART at a CD4 cell count of 350/µl. Simulations using a higher CD4 cell count threshold for treatment and/or a greater number of contemporary treatment regimens are likely to project even longer life expectancies.

Exercises that involve iterative assessment of model performance can provide information about the relative influence of different uncertain assumptions, illuminate unexpected synergies between parameters, and provide insight into particular heterogeneities within and between cohorts. When data are available to allow for exercises like those described here, they can be used to assess model performance; descriptive analyses of the process taken to do so can contribute to a dialogue about different approaches that are taken by analysts to assess model process and model structure uncertainty.

## Supporting Information

Supporting Information S1 Supplementary tables and figures referenced in the main text are provided.
(1.27 MB PDF)
