# Supplementary material for: Assessing the Performance of a Computer-Based Policy Model of HIV and AIDS
Source: PLoS One. 2010 Sep 9;5(9):e12647. doi: 10.1371/journal.pone.0012647 (PMC2936574; doi:10.1371/journal.pone.0012647)
Supplement: Supporting Information S1 — Supplementary tables and figures referenced in the main text are provided. (1.27 MB PDF) [file pone.0012647.s001.pdf]

Supporting Information

Accompanying the manuscript:

Assessing the Performance of a Computer-Based Policy Model of HIV and AIDS

Chara E. Rydzak, BA
Kara L. Cotich, BS
Paul E. Sax, MD
Heather E. Hsu, MPH
Bingxia Wang, PhD
Elena Losina, PhD
Kenneth A. Freedberg, MD, MSc
Milton C. Weinstein, PhD
Sue J. Goldie, MD, MPH
For the CEPAC Investigators

Table of Contents:

Table S1. Select base case model parameter inputs derived from the WIHS
Pg. 3-6

Table S2. Select base case model parameter inputs derived from the MACS
Pg. 7-8

Table S3. Selected treatment parameter values
Pg. 9-11

Table S4. Summary of selected treatment parameter values of contemporary regimens
Pg. 12-13

Table S5. Summary table of changes in 'clinical effectiveness' of HAART'
Pg. 14-16

Figure S1A. Model-projected versus WIHS empiric 24-month survival (CD4 50-199/µl): Examples of selected one-way sensitivity analyses.

Pg. 17

Figure S1B. Model-projected versus WIHS empiric 24-month survival (CD4 50-199/µl): Examples of selected multi-way sensitivity analyses.

Pg. 18

Table S1. Select base case model parameter inputs derived from the WIHS

Parameter	Base Case Value (SD)	Range	Source
**Cohort Characteristics**			
Mean Age (SD)	36.1 yrs (8)		[31]
**Initial mean CD4 cell count (SD)/µl**			[31]
Cohort Mean	520.40 (417.50)		
CD4<50	19.43 (14.71)		
CD4 50-199	128.76 (43.01)		
CD4 200-349	274.20 (42.62)		
CD4 ≥350	602.43 (226.07)		
**HIV Viral Load Distribution (copies/µl)**			[31]
**Cohort Mean**			
Viral Load >100,000	0.26171		
Viral Load 30,000-100,000	0.19705		
Viral Load 10,000-30,000	0.17617		
Viral Load 3,000-10,000	0.25305		
Viral Load 500-3,000	0.06619		
Viral Load <500	0.04582		
**CD4<50/µl**			
Viral Load >100,000	0.68229		
Viral Load 30,000-100,000	0.19271		
Viral Load 10,000-30,000	0.08854		
Viral Load 3,000-10,000	0.03125		
Viral Load 500-3,000	0.00521		
Viral Load <500	0		
**CD4 50-199/µl**			
Viral Load >100,000	0.48232		
Viral Load 30,000-100,000	0.25402		
Viral Load 10,000-30,000	0.12862		
Viral Load 3,000-10,000	0.08360		
Viral Load 500-3,000	0.03537		
Viral Load <500	0.01608		
**CD4 200-349/µl**			
Viral Load >100,000	0.24235		
Viral Load 30,000-100,000	0.25765		
Viral Load 10,000-30,000	0.18878		
Viral Load 3,000-10,000	0.22704		
Viral Load 500-3,000	0.04847		
Viral Load <500	0.03571		
**CD4 ≥350/µl**			
Viral Load >100,000	0.09278		
Viral Load 30,000-100,000	0.14204		
Viral Load 10,000-30,000	0.21879		
Viral Load 3,000-10,000	0.36884		
Viral Load 500-3,000	0.09737		

Viral Load <500	0.08018		
**Natural History Parameters**			
**Monthly Probability of Chronic AIDS Death***			[31]
**No History of OI (CD4/µl)**			
CD4 >500	0.000833	0-0.000833	
CD4 300-500	0.000833	0-0.000833	
CD4 200-300	0.000833	0-0.000833	
CD4 100-200	0.00416	0.00149-0.00416	
CD4 50-100	0.00416	0.000833-0.00416	
CD4 <50	0.01653	0.00995-0.01653	
**History of OI (CD4/µl)**			
CD4 >500	0.00664	0.00250-0.00664	
CD4 300-500	0.00995	0.00499-0.00995	
CD4 200-300	0.01571	0.00830-0.01571	
CD4 100-200	0.02388	0.01571-0.02388	
CD4 50-100	0.03439	0.02062-0.03439	
CD4 <50	0.06137	0.05194-0.06137	
**Monthly CD4 Decline After Treatment Failure copies/µl (SD)**			[31]
Viral Load >100,000	2.93 (0.42)	1.465-6.375	
Viral Load 30,000-100,000	2.61 (0.38)	1.305-6.375	
Viral Load 10,000-30,000	2.59 (0.15)	1.295-5.40	
Viral Load 3,000-10,000	2.59 (0.15)	1.240-4.60	
Viral Load 500-3,000	2.48 (0.20)	1.24-3.733	
Viral Load <500	2.48 (0.20)	1.24-3.025	
**Monthly Probabilities of OI (CD4/µl)**			[31]
**PCP**			
CD4 >500	0.00083	0-0.00083	
CD4 300-500	0.00083	0-0.00085	
CD4 200-300	0.00250	0.000833-0.00373	
CD4 100-200	0.00416	0.001665-0.0096	
CD4 50-100	0.00995	0.002497-0.031	
CD4 <50	0.01489	0.006644-0.037	
**MAC**			
CD4 >500	0.00017	0-0.00017	
CD4 300-500	0.00017	0-0.00017	
CD4 200-300	0.00042	0-0.00042	
CD4 100-200	0.00042	0-0.00101	
CD4 50-100	0	0-0.00375	
CD4 <50	0.01143	0.004988-0.0122	
**Toxoplasmosis**			
CD4 >500	0.00008	0-0.00008	
CD4 300-500	0	0-0.00009	
CD4 200-300	0.00042	0-0.00042	
CD4 100-200	0.00042	0-0.00067	

CD4 50-100	0.00250	0-0.00250	
CD4 <50	0.00183	0-0.0027	
**CMV**			
CD4 >500	0.00017	0-0.00017	
CD4 300-500	0.00033	0-0.00033	
CD4 200-300	0.00017	0-0.00058	
CD4 100-200	0.00042	0-0.00214	
CD4 50-100	0.00250	0-0.00523	
CD4 <50	0.00374	0.00083-0.01857	
**Fungal Infection**			
CD4 >500	0.00083	0-0.00083	
CD4 300-500	0.00167	0.00833-0.00167	
CD4 200-300	0.00333	0.00167-0.00333	
CD4 100-200	0.00333	0.00083-0.00333	
CD4 50-100	0.01077	0.00250-0.01077	
CD4 <50	0.02469	0.01407-0.02469	
**Bacterial Infection**			
CD4 >500	0.00167	0-0.00167	
CD4 300-500	0.00250	0-0.00250	
CD4 200-300	0.00416	0-0.00416	
CD4 100-200	0.00333	0-0.00333	
CD4 50-100	0.00167	0-0.00167	
CD4 <50	0.00664	0-0.00664	
**Invasive Cervical Cancer**			
CD4 >500	0.00042	0-0.00042	
CD4 300-500	0.00083	0-0.00083	
CD4 200-300	0.00017	0-0.00017	
CD4 100-200	0.00042	0-0.00042	
CD4 50-100	0.00258	0-0.00258	
CD4 <50	0.00183	0-0.00183	
**Other OI**			
CD4 >500	0.00582	0.00416-0.00582	
CD4 300-500	0.00499	0.00333-0.00499	
CD4 200-300	0.00582	0.00333-0.00582	
CD4 100-200	0.01077	0.00582-0.01077	
CD4 50-100	0.01653	0.00582-0.0246	
CD4 <50	0.01898	0.00830-0.0394	
**Monthly Probabilities of Acute Death from OI**			[31]
PCP	0.007	0.00035-0.0632	
MAC	0.026	0.0130-0.1585	
Toxoplasmosis	0	0-0.1786	
CMV	0	0-0.1053	
Fungal Infection	0.003	0.0015-0.0566	
Bacterial Infection	0.012	0-0.0120	
Invasive Cervical Cancer	0	0	

Other OI	0.007	0.0035-0.0749

CMV = Cytomegalovirus; MAC = *Mycobacterium avium* complex; OI = opportunistic infection(s); PCP = formerly *Pneumocystis carinii* pneumonia, now renamed *Pneumocystis jiroveci* pneumonia; WIHS = Women's Interagency HIV Study

*These values reflect initial mean estimates of chronic AIDS mortality given a woman's OI history. Assessment of the performance of the natural history model demonstrated that using lower estimates of the mean values for CD4 >50/µl provided better estimates of the empiric survival. These values were subsequently adjusted and applied to analyses of the treatment model. Adjusted values of monthly chronic AIDS mortality given a history of previous OI for CD4 50-199/µl were decreased by 25%; for example, 0.00664 becomes 0.00498. Adjusted values of monthly chronic AIDS mortality given a history of previous OI for CD4 >200/µl were decreased by 50%; for example, 0.00664 becomes 0.00332.

Table S2. Select base case model parameter inputs derived from the MACS

Parameter	Base Case (SD)	Source
**Cohort Characteristics**		
Mean Age (SD)	33 yrs	[35]
**Initial mean CD4 cell count (SD)/µl**		[35]
Cohort Mean	588 (274)	
**HIV Viral Load Distribution (copies/µl)**		[35]
**Cohort Mean**		
Viral Load >100,000	0	
Viral Load 30,000-100,000	0.257	
Viral Load 10,000-30,000	0.250	
Viral Load 3,000-10,000	0.252	
Viral Load 500-3,000	0.163	
Viral Load <500	0.077	
**Natural History Parameters**		
**Monthly Probability of Chronic AIDS Death**		[35]
**No History of OI (CD4/µl)**		
CD4 >500	0.000063	
CD4 300-500	0.000094	
CD4 200-300	0.00106	
CD4 100-200	0.00149	
CD4 50-100	0.00861	
CD4 <50	0.01853	
**History of OI (CD4/µl)**		
CD4 >500	0.0030	
CD4 300-500	0.0170	
CD4 200-300	0.0274	
CD4 100-200	0.02145	
CD4 50-100	0.02332	
CD4 <50	0.09682	
**Monthly CD4 Decline After Treatment Failure/µl**		[35]
Viral Load >100,000	6.375	
Viral Load 30,000-100,000	6.375	
Viral Load 10,000-30,000	5.40	
Viral Load 3,000-10,000	4.60	
Viral Load 500-3,000	3.733	
Viral Load <500	3.025	
**Monthly Probabilities of OI (CD4/µl)**		[35]
**PCP**		
CD4 >500	0.00041	
CD4 300-500	0.00085	
CD4 200-300	0.00373	
CD4 100-200	0.0096	
CD4 50-100	0.0310	
CD4 <50	0.0370	
**MAC**		

CD4 >500	0.000059	
CD4 300-500	0.000055	
CD4 200-300	0.00022	
CD4 100-200	0.00101	
CD4 50-100	0.00375	
CD4 <50	0.01220	
**Toxoplasmosis**		
CD4 >500	0.000029	
CD4 300-500	0.000092	
CD4 200-300	0.00042	
CD4 100-200	0.00067	
CD4 50-100	0.00140	
CD4 <50	0.00270	
**CMV**		
CD4 >500	0.000059	
CD4 300-500	0.000129	
CD4 200-300	0.00058	
CD4 100-200	0.00214	
CD4 50-100	0.00523	
CD4 <50	0.01857	
**Fungal Infection**		
CD4 >500	0.000088	
CD4 300-500	0.000276	
CD4 200-300	0.00029	
CD4 100-200	0.00135	
CD4 50-100	0.00591	
CD4 <50	0.01123	
**Other OI**		
CD4 >500	0.00047	
CD4 300-500	0.00087	
CD4 200-300	0.0024	
CD4 100-200	0.0076	
CD4 50-100	0.0246	
CD4 <50	0.0394	
**Monthly Probabilities of Acute Death from OI**		[35]
PCP	0.0632	
MAC	0.1585	
Toxoplasmosis	0.1786	
CMV	0.1053	
Fungal Infection	0.0566	
Bacterial Infection	0	
Invasive Cervical Cancer	0	
Other OI	0.0749	

CMV = Cytomegalovirus; MAC = *Mycobacterium avium* complex; MACS = Multicenter AIDS Cohort Study; OI = opportunistic infection(s); PCP = formerly *Pneumocystis carinii* pneumonia, now renamed *Pneumocystis jiroveci* pneumonia

Table S3. Selected treatment parameter values

Parameter	Base Case Value	Range	Source
**Cohort Treatment Characteristics**			
**Cohort Racial Composition (n)**			[33]
African American	573		
White	184		
Hispanic	204		
**ART Naïve Prior to HAART Initiation (%)**			[33]
African American	15.5		
White	19.6		
Hispanic	19.6		
**Pre-HAART Peak HIV RNA (copies/µl)**			[33]
African American	83,000		
White	78,500		
Hispanic	78,000		
**Pre-HAART Nadir CD4/µl**			[33]
African American	199		
White	214		
Hispanic	216		
**Pre-HAART Diagnosis of AIDS (%)**			[33]
African American	47.5		
White	43.5		
Hispanic	44.6		
**HAART Treatment Parameters**			
**CD4 Cell Count/µl Threshold to Start HAART**	200	200-350	
**Regimen Efficacy (% Viral Load Suppression at 24 weeks)***			[42,46-49]
1. EFV + AZT + 3TC	75.0%	7.5%-100%	
2. IDV + AZT (or d4T) + 3TC	60.0%	6%-90%	
3. LPV/r + TDF + FTC + AZT	61.0%	6%-91%	
4. ENF + OBR	32.7%	3%-49%	
5. OBR (2 PIs + 2 NRTIs)	15.0%	1%-23%	
**Pooled Monthly Probability of Late Treatment Failure†**	0.021099	0.0086-0.0439	[42,46-49]
**Mean CD4/µl Gain on Successful Treatment over the First 12 Months of Treatment (SD)**			[42,46-49]
**Time Period 1 (months 1-2)**			
1. EFV + AZT + 3TC	68.81 (17.20)	6.88-86.01	
2. IDV + AZT (or d4T) + 3TC	25.02 (6.26)	2.50-31.28	
3. LPV/r + TDF + FTC + AZT	68.71 (17.18)	6.87-85.89	
4. ENF + OBR	75.63 (18.91)	7.56-94.55	
5. OBR (2 PIs + 2 NRTIs)	26.04 (6.51)	2.60-32.55	

**Time Period 2 (months 3-12)**			
1. EFV + AZT + 3TC	3.60 (0.90)	0.36-4.50	
2. IDV + AZT (or d4T) + 3TC	1.31 (0.33)	0.13-1.64	
3. LPV/r + TDF + FTC + AZT	3.60 (0.90)	0.36-4.50	
4. ENF + OBR	3.96 (0.99)	0.40-4.95	
5. OBR (2 PIs + 2 NRTIs)	1.36 (0.34)	0.14-1.70	
**ART Effect‡**			[50,51]
CD4 ≤50/µl	0.78	0.54-1.0	
CD4 >50/µl	0.66	0.54-1.0	
**OI Treatment Parameters**			
**Efficacy of OI Prophylaxis**			[67-69]
**PCP**			
TMP-SMX 7DS	0.996		
Dapsone	0.872		
Aerosolized Pentamidine	0.650		
**MAC**			
Azithromycin	0.772		
Clarithromycin	0.759		
Rifabutin	0.570		
**Toxoplasmosis**			
TMP-SMX 7DS	0.650		
Pyrimethamine/Leucovorin	0.650		
**Major Toxicity Associated with OI Prophylaxis**			[67-69]
**PCP**			
TMP-SMX 7DS	0.2320		
Dapsone	0.2028		
Aerosolized Pentamidine	0.0250		
**MAC**			
Azithromycin	0.130		
Clarithromycin	0.1398		
Rifabutin	0.160		
**Toxoplasmosis**			
TMP-SMX 7DS	0		
Pyrimethamine/Leucovorin	-		

3TC = lamivudine; ART = antiretroviral therapy; AZT = zidovudine; ddI = didanosine; CMV = Cytomegalovirus; d4T = stavudine; EFV = efavirenz; ENF = enfuvirtide; FTC = emtricitabine; HAART = highly active antiretroviral therapy; IDV = indinavir; LPV/r = lopinavir/ritonavir; MAC = *Mycobacterium avium* complex; NRTI = nucleoside reverse transcriptase inhibitors; NNRTI = non-nucleoside reverse transcriptase inhibitors; OBR = optimized background antiretroviral regimen; OI = opportunistic infection(s); PCP = formerly *Pneumocystis carinii* pneumonia , now renamed *Pneumocystis jiroveci* pneumonia; TDF = tenofovir; TMP-SMX = trimethoprim and sulfamethoxazole (Bactrim); WIHS = Women's Interagency HIV Study

* Base case analyses used the following four regimens, respectively, Regimen 1 (EFV + AZT + 3TC), Regimen 2 (IDV + AZT (or d4T) + 3TC), Regimen 3 (LPV/r + TDF + FTC + AZT), and

Regimen 5 (OBR (2 PIs + 2 NRTIs)). Sensitivity analyses performed using 5 lines of treatment used the regimens listed in the table in the order in which they are presented with the fourth regimen of ENF + OBR followed by Regimen 5 (OBR (2 PIs + 2 NRTIs)). Later regimens differ slightly between the base case 4-regimen scenario compared to the 5-regimen scenario as we assumed that more current regimens would become available to women if they survived more than 12-24 months. The order of regimens reflects the general sequence in which regimens are used. HAART was assumed to be initiated when CD4 cell count reached 200/µl given this was the standard practice during the cohort timeframe over which treatment occurred and survival data was collected.

† The probability of late failure is the monthly risk of failing treatment after initial successful viral load suppression at 24 weeks. The estimate is based on a pooled weighted average using reported data from the studies of the HAART regimens used in the analysis.

‡ The ART effect is defined as an independent protective effect of HAART and is modeled as a multiplier which decreases the incidence of opportunistic infections and AIDS-related mortality in patients with virologic failure who remain on HAART. (See methods for details)

Table S4. Summary of selected treatment parameter values of contemporary regimens

Variable or Assumption	Parameter Value	Source
**HAART Efficacy (% Viral Load Suppression at 24 weeks)**		
EFV + 2 NRTIs	86.0%	[52,54]
ATV/RTV + 2 NRTIs‡	79.0%	[47,48]
RAL + DRV/RTV + OBR	77.5%	[55]
T20 + OBR or MVC + OBR +/-T20||	40.0%	[46,53]
OBR (2 PIs + 2 NRTIs)	15.0%	[46]
**Mean CD4 Gain/µl on Successful Treatment over first 12 months (SD)**		
**Time Period 1 (months 1-2)**		
EFV + 2 NRTIs	90.03 (22.51)	[52,54]
ATV/RTV + 2 NRTIs‡	43.98 (10.99)	[47,48]
RAL + DRV/RTV + OBR	56.82 (14.20)	[55]
T20 + OBR or MVC + OBR +/- T20||	77.72 (19.43)	[46,53]
OBR (2 PIs + 2 NRTIs)	26.04 (6.51)	[46]
**Time Period 2 (months 3-12)**		
EFV + 2 NRTIs	4.67 (1.17)	[52,54]
ATV/RTV + 2 NRTIs‡	2.28 (0.57)	[47,48]
RAL + DRV/RTV + OBR	2.95 (0.74)	[55]
T20 + OBR or MVC + OBR +/- T20||	4.08 (1.02)	[46,53]
OBR (2 PIs + 2 NRTIs)	1.36 (0.34)	[46]
**Late Treatment Failure***		
Pooled Monthly Probability	0.0184	[46-48,52-55]
**ART Effect†**		
CD4 <50/µl	0.78	[50]
CD4 >50/µl	0.66	[50]
**Assumptions**		
Delay in CD4 decline	1 year	
Force failure	10 years	

ART = antiretroviral therapy; ATV = atazanavir; DRV = darunavir; EFV = efavirenz; HAART = highly active antiretroviral therapy; MVC = maraviroc; NRTI = nucleoside reverse transcriptase inhibitors; NNRTI = non-nucleoside reverse transcriptase inhibitors; OBR = optimized background antiretroviral regimen; PI = protease inhibitor; RAL = raltegravir; RTV = ritonavir; SD = standard deviation; T20 = enfuvirtide

* The probability of late failure is the monthly risk of failing treatment after initial successful viral load suppression at 24 weeks.

† The ART effect is defined as an independent protective effect of HAART and is modeled as a multiplier which decreases the incidence of opportunistic infections and AIDS-related mortality in patients with virologic failure who remain on HAART.

‡ ATV/r arm, <4 PI mutations

§ Although the virologic suppression for this regimen was reported at 16 weeks, the model treats it as 24 week data.

|| We assumed that 50% of the cohort received T20 + OBR (HIV RNA 32.7% suppressed at 24 weeks; CD4 benefit of 119 cells at 48 weeks27) and 50% received MVC + OBR +/- T20 (BID arm: HIV RNA 60.4% suppressed at 24 weeks and a CD4 benefit of 111 cells at 24 weeks25; 46% of these patients received T20). The viral suppression rate and the CD4 benefits reported in this table are weighted averages of these two regimens.

Table S5. Summary table of changes in 'clinical effectiveness' of HAART'

	Ultimate proportion suppressed in the cohort given HAART efficacy, adherence, toxicity, and personal choice to continue HAART	'Clinical Effectiveness' (function of efficacy, adherence, toxicity, personal choice to continue HAART)	'Implied failure,' discontinuation, or switching of HAART in the cohort	Factor increase in 'implied failure,' discontinuation, or switching of HAART
Line 1	EFV + AZT + 3TC: Proportion suppressed in clinical trial = 75% (corresponding base case failure = 25%)			
	75% decrease	18.75%	81.25%	3.25
	70% decrease	22.50%	77.50%	3.10
	60% decrease	30.00%	70.00%	2.80
	50% decrease	37.50%	62.50%	2.50
	40% decrease	45.00%	55.00%	2.20
	30% decrease	52.50%	47.50%	1.90
	Base Case	75.00%	25.00%	1.00
Line 2	IDV + AZT (or d4T) + 3TC: Proportion suppressed in clinical trial = 60% (corresponding base case failure = 40%)			
	75% decrease	15.00%	85.00%	2.13
	70% decrease	18.00%	82.00%	2.05
	60% decrease	24.00%	76.00%	1.90
	50% decrease	30.00%	70.00%	1.75
	40% decrease	36.00%	64.00%	1.60
	30% decrease	42.00%	58.00%	1.45
	Base Case	60.00%	40.00%	1.00
	**Ultimate proportion suppressed in the cohort given HAART efficacy, adherence, toxicity, and personal choice to continue HAART**	**'Clinical Effectiveness' (function of efficacy, adherence, toxicity, personal choice to continue HAART)**	**'Implied failure,' discontinuation, or switching of HAART in the cohort**	**% Reduction in implied failure of HAART**
Line 3	LPV/r + TDF + FTC + AZT: proportion suppressed in clinical trial = 61% (corresponding base case failure = 39%)			
	60% increase†	95.00%	5.00%	87.18%
	50% increase	91.50%	8.50%	78.21%

	40% increase	85.40%	14.60%	62.56%
	30% increase	79.30%	20.70%	46.92%
	Base Case	61.00%	39.00%	0.00%
Line 4	OBR (2 PIs + 2 NRTIs): proportion suppressed in clinical trial = 15% (corresponding base case failure = 85%)			
	75% increase	26.25%	73.75%	13.24%
	70% increase	25.50%	74.50%	12.35%
	60% increase	24.00%	76.00%	10.59%
	50% increase	22.50%	77.50%	8.82%
	40% increase	21.00%	79.00%	7.06%
	30% increase	19.50%	80.50%	5.29%
	Base Case	15.00%	85.00%	0%
	**Ultimate proportion suppressed in the cohort given HAART efficacy, adherence, toxicity, and personal choice to continue HAART**	**'Clinical Effectiveness' (function of efficacy, adherence, toxicity, personal choice to continue HAART)**	**'Implied failure,' discontinuation, or switching of HAART in the cohort**	**% Reduction in implied failure of HAART**
	Sensitivity Analysis – 5 lines of HAART Available			
Line 4 of 5	ENF + OBR: proportion suppressed in clinical trial = 33% (corresponding base case failure = 66%)			
	75% increase	57.75%	42.25%	36.94%
	70% increase	56.10%	43.90%	34.48%
	60% increase	52.80%	47.20%	29.55%
	50% increase	49.50%	50.50%	24.63%
	40% increase	46.20%	53.80%	19.70%
	30% increase	42.90%	57.10%	14.78%
	Base Case	32.70%	67.00%	0%
Line 5 of 5	OBR (2 PIs + 2 NRTIs): proportion suppressed in clinical trial = 15% (corresponding base case failure = 85%)			
	75% increase	26.25%	73.75%	13.24%
	70% increase	25.50%	74.50%	12.35%
	60% increase	24.00%	76.00%	10.59%
	50% increase	22.50%	77.50%	8.82%

40% increase	21.00%	79.00%	7.06%
30% increase	19.50%	80.50%	5.29%
Base Case	15.00%	85.00%	0%

3TC = lamivudine; ART = antiretroviral therapy; AZT = zidovudine; ddI = didanosine; d4T = stavudine; EFV = efavirenz; ENF = enfuvirtide; FTC = emtricitabine; HAART = highly active antiretroviral therapy; IDV = indinavir; LPV/r = lopinavir/ritonavir; NRTI = nucleoside reverse transcriptase inhibitors; NNRTI = non-nucleoside reverse transcriptase inhibitors; OBR = optimized background antiretroviral regimen; TDF = tenofovir

A

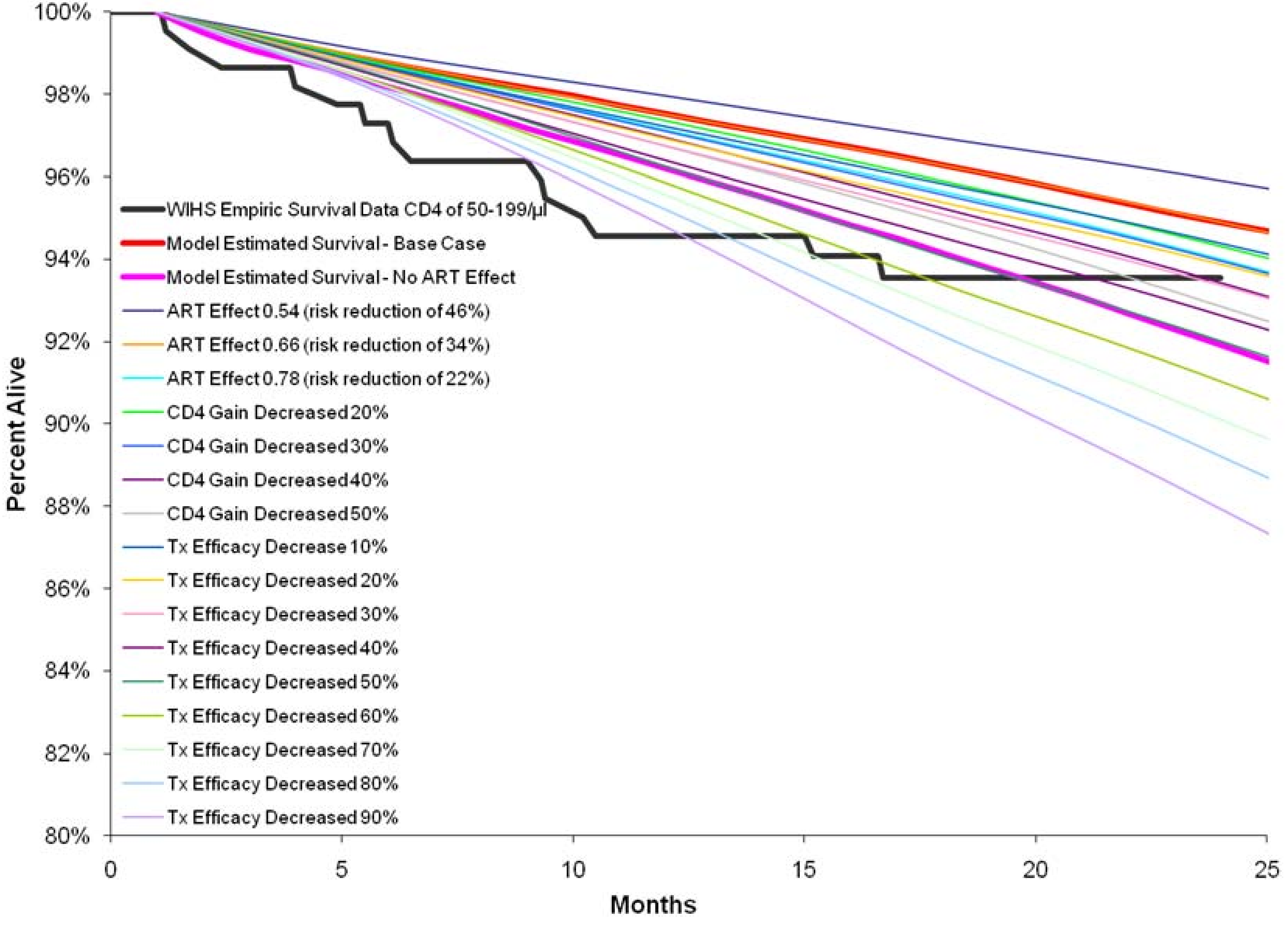

WIHS Empiric Survival Data CD4 of 50-199/µl
Model Estimated Survival - Base Case
Model Estimated Survival - No ART Effect
ART Effect 0.54 (risk reduction of 46%)
ART Effect 0.66 (risk reduction of 34%)
ART Effect 0.78 (risk reduction of 22%)
CD4 Gain Decreased 20%
CD4 Gain Decreased 30%
CD4 Gain Decreased 40%
CD4 Gain Decreased 50%
Tx Efficacy Decrease 10%
Tx Efficacy Decreased 20%
Tx Efficacy Decreased 30%
Tx Efficacy Decreased 40%
Tx Efficacy Decreased 50%
Tx Efficacy Decreased 60%
Tx Efficacy Decreased 70%
Tx Efficacy Decreased 80%
Tx Efficacy Decreased 90%
Percent Alive
100%
98%
96%
94%
92%
90%
88%
86%
84%
82%
80%
0
5
10
15
20
25
Months

B

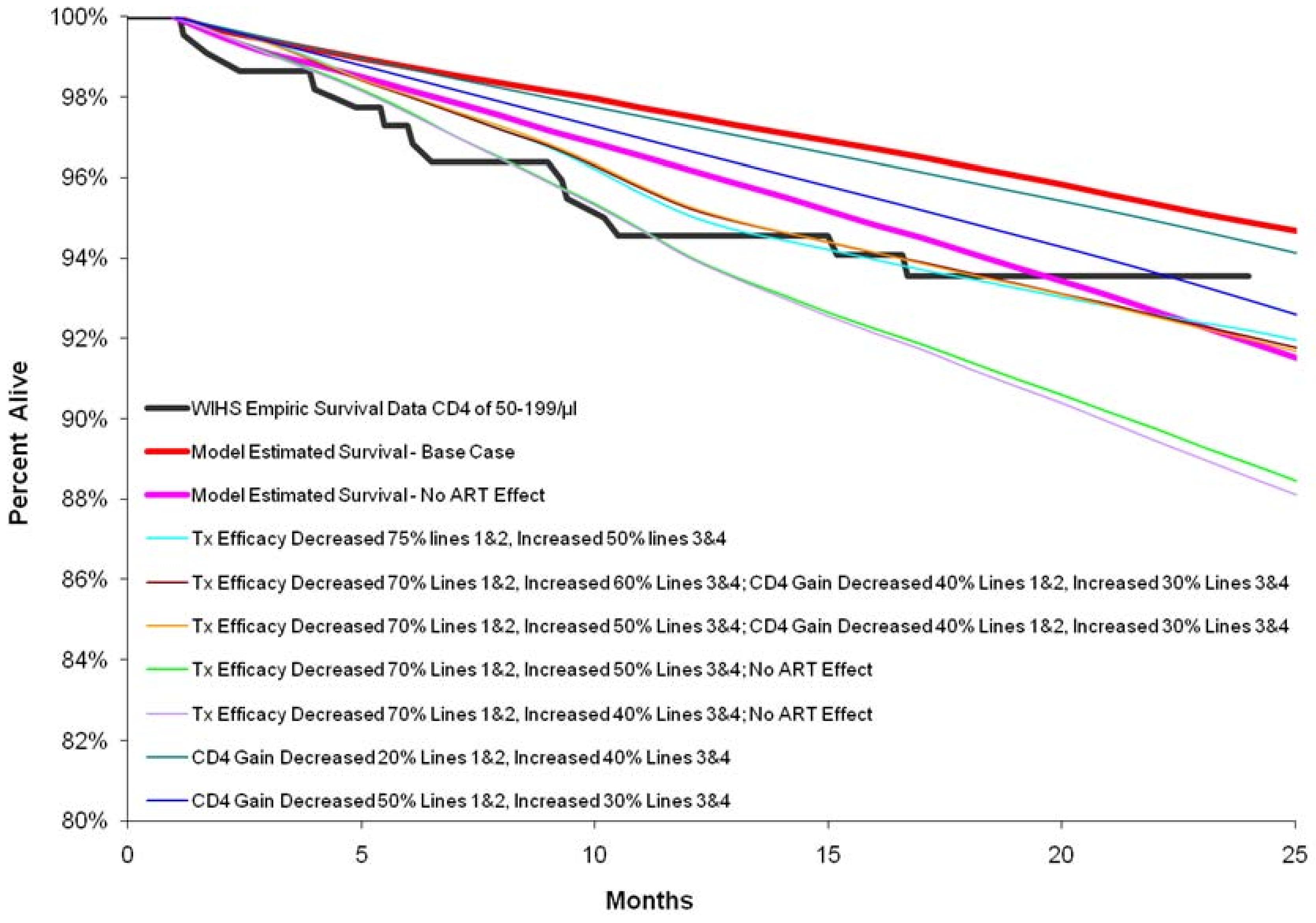

100%
98%
96%
94%
92%
90%
88%
86%
84%
82%
80%
Percent Alive
0
5
10
15
20
25
Months
WIHS Empiric Survival Data CD4 of 50-199/µl
Model Estimated Survival - Base Case
Model Estimated Survival - No ART Effect
Tx Efficacy Decreased 75% lines 1&2, Increased 50% lines 3&4
Tx Efficacy Decreased 70% Lines 1&2, Increased 60% Lines 3&4; CD4 Gain Decreased 40% Lines 1&2, Increased 30% Lines 3&4
Tx Efficacy Decreased 70% Lines 1&2, Increased 50% Lines 3&4; CD4 Gain Decreased 40% Lines 1&2, Increased 30% Lines 3&4
Tx Efficacy Decreased 70% Lines 1&2, Increased 50% Lines 3&4; No ART Effect
Tx Efficacy Decreased 70% Lines 1&2, Increased 40% Lines 3&4; No ART Effect
CD4 Gain Decreased 20% Lines 1&2, Increased 40% Lines 3&4
CD4 Gain Decreased 50% Lines 1&2, Increased 30% Lines 3&4

SUPPORTING INFORMATION – FIGURE LEGENDS

Figure S1A-B. Model-projected versus WIHS empiric 24-month survival for CD4 50-199/µl: Examples of selected one-way and multi-way sensitivity analyses. Part A of Figure S1 illustrates a subset of one-way sensitivity analyses performed in which treatment efficacy, CD4 gain estimates and ART effect were individually varied. These variables were most influential in reducing the model-projected survival although generally did not achieve simultaneous consistency with both 12 and 24 month survival. Scenarios most consistent with the empiric data at 12 months underestimated survival at 24 months while those most consistent with the empiric data at 24 months overestimated survival at 12 months. Part B of Figure S1 illustrates a sample of multi-way sensitivity analyses that were performed. Using insights from the one-way sensitivity analyses, multi-way sensitivity analyses allowed assumptions to vary by regimen (e.g., decrease in efficacy for 1st and 2nd line ART but an increase in efficacy for 3rd and 4th line ART) and also allowed changes in two or more variables simultaneously (e.g., decrease in efficacy ART and decrease in CD4 gain).
